# Transport of Anthocyanins and other Flavonoids by the Arabidopsis ATP-Binding Cassette Transporter AtABCC2

**DOI:** 10.1038/s41598-018-37504-8

**Published:** 2019-01-24

**Authors:** Claire E. Behrens, Kaila E. Smith, Cristina V. Iancu, Jun-yong Choe, John V. Dean

**Affiliations:** 1 Department of Biological Sciences, DePaul University, 2325 N. Clifton Ave., Chicago, 60614 IL USA; 2 Department of Biochemistry and Molecular Biology, Rosalind Franklin University of Medicine and Science, 3333 Green Bay Road, North Chicago, 60064 IL USA; 3 Present Address: East Carolina Diabetes and Obesity Institute, East Carolina University, Greenville, NC 27834 USA

## Abstract

Flavonoids have important developmental, physiological, and ecological roles in plants and are primarily stored in the large central vacuole. Here we show that both an ATP-binding cassette (ABC) transporter(s) and an H^+^-antiporter(s) are involved in the uptake of cyanidin 3-*O*-glucoside (C3G) by Arabidopsis vacuolar membrane-enriched vesicles. We also demonstrate that vesicles isolated from yeast expressing the ABC protein AtABCC2 are capable of MgATP-dependent uptake of C3G and other anthocyanins. The uptake of C3G by AtABCC2 depended on the co-transport of glutathione (GSH). C3G was not altered during transport and a GSH conjugate was not formed. Vesicles from yeast expressing AtABCC2 also transported flavone and flavonol glucosides. We performed ligand docking studies to a homology model of AtABCC2 and probed the putative binding sites of C3G and GSH through site-directed mutagenesis and functional studies. These studies identified residues important for substrate recognition and transport activity in AtABCC2, and suggest that C3G and GSH bind closely, mutually enhancing each other’s binding. In conclusion, we suggest that AtABCC2 along with possibly other ABCC proteins are involved in the vacuolar transport of anthocyanins and other flavonoids in the vegetative tissue of Arabidopsis.

## Introduction

Flavonoids are a large group of phenolic compounds that are present in most plant tissue. The three major classes of flavonoids include the red to purple anthocyanins, the pale yellow flavonols, and the proanthocyanidins (PA, condensed tannins) of seed coats. Of these, the anthocyanins represent the largest class^1^. Flavonoids have important developmental, physiological and ecological roles in plants including control of auxin transport, allelopathy, UV protection, biotic and abiotic defense, and the attraction of pollinators and seed dispersers^2^. In addition, there is a great deal of interest in these compounds due to their human health-promoting effects^3,4^. Flavonoids are produced on the cytosolic side of the endoplasmic reticulum and then accumulate in most plant cell compartments. However, the conjugated anthocyanins, and flavonol and flavone glycosides primarily accumulate in the vacuole^5^. In addition, monomers such as catechin and epicatechin are glucosylated and transported into the vacuole where they polymerize into PA in the endothelial layer of the seed coat and are eventually secreted into the apoplast^6^. Though the biosynthesis and regulation of flavonoid formation is one of the most intensively studied biochemical pathways in plants, the events leading to the vacuolar sequestration of these compounds remain largely unknown.

The two general mechanisms described for the vacuolar transport of flavonoids include membrane transporter- and vesicle trafficking-mediated transport^7,8^. These two mechanisms are likely not mutually exclusive and, depending on the species, may both be involved in vacuolar flavonoid transport^8^. Both mechanisms appear to require the assistance of a glutathione *S*-transferase (GST)^9–11^. However, none of these GSTs are able to conjugate glutathione (GSH) to anthocyanins, and GSH conjugates of anthocyanins have never been found to exist in plants^10^. Once synthesized, flavonoids undergo a variety of chemical modifications including glycosylation, methylation and acylation^12^, however, glycosylation alone is thought to be sufficient for recognition by vacuolar transporters^5^. There are typically two main membrane transport mechanisms proposed for the vacuolar uptake of glycosylated phenolic compounds. These include H^+^-antiport and uptake by ATP-binding cassette (ABC) transporters^13,14^.

**Figure 1:**
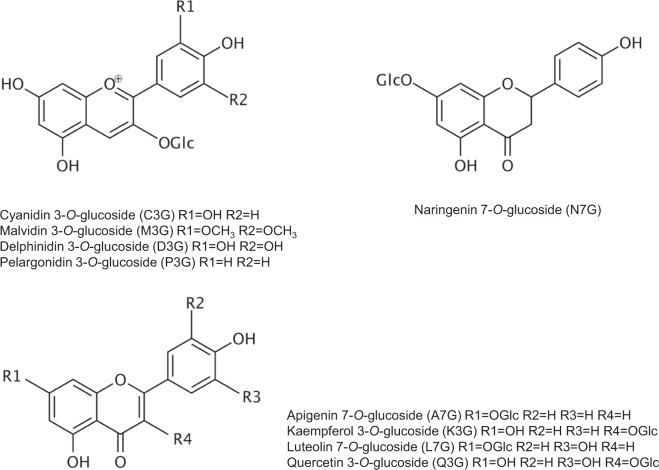
Diagram of anthocyanins and other flavonoids described in this study.

The multidrug and toxic compound extrusion (MATE) transporters appear to be the H^+^-antiporters involved in the vacuolar transport of flavonoids. There are 58 MATE transporters encoded in the genome of Arabidopsis. However, very few of these have been functionally characterized^15^. The involvement of MATE transporters in the vacuolar uptake of flavonoids was first determined through the characterization of TRANSPARENT TESTA12 (TT12). TT12 is a MATE transporter localized to the vacuolar membrane and mechanistically active as a flavonoid glucoside/H^+^-antiporter in PA synthesizing cells of the seed coat^16,17^. The role of MATE proteins in the vacuolar accumulation of PA precursors or anthocyanins has also been demonstrated in *Medicago truncatula*^6,18^, tomato^19^ and grapevine^20^. TT12, MtMATE1 and MtMATE2, have all been shown to transport cyanidin 3-*O*-glucoside (C3G; Fig. 1) when heterologously expressed in yeast^6,17,18^.

The plant vacuolar membrane also contains ABC transporters that move large organic anions into the vacuole^21–23^. ABC transporters are directly energized by ATP hydrolysis and therefore do not depend on the transmembrane H^+^-electrochemical gradient. ABC transporters are characteristically inhibited by vanadate, a phosphoryl transition-state analogue. There are several lines of evidence that ABC transporters are involved in the vacuolar uptake of flavonoids, specifically, the ABCC subclass (previously known as multidrug resistance-associated proteins [MRP]). The vacuolar uptake of flavone glucuronide conjugates by rye mesophyll vacuoles and the uptake of a flavone glucoside by Arabidopsis vacuoles were shown to occur through an ABC transporter-type mechanism^13,24^. However, in both cases, a specific transporter was not identified and in the case of Arabidopsis, the flavone glucosides examined were not synthesized by this species. Genetic studies in maize provided evidence that ZmABCC3 (formerly ZmMRP3) was involved in the vacuolar uptake of C3G^25^. ZmABCC4 (formerly ZmMRP4), a homolog of ZmABCC3, was thought to be responsible for the vacuolar localization of anthocyanins in the aleurone^25^. However, it has never been demonstrated that ZmABCC3 or ZmABCC4 can functionally transport C3G *in vitro*. More recently, it has been shown that the ABC transporter VvABCC1, is localized to the tonoplast of grape berry exocarp cells and is biochemically capable of co-transporting anthocyanidin 3-*O*-glucosides and GSH when heterologously expressed in yeast^26^. The co-transport of GSH with bile acids, vincristine, and other drugs by a human ABCC transporter has also been previously demonstrated^27–29^.

Though it is well known that TT12 is a MATE transporter responsible for the transport of flavonoids into the seed coat endothelium of Arabidopsis seeds^16,17^, and that TT12 can also transport anthocyanins when heterologously expressed in yeast^6,17^, the transporter(s) involved in vacuolar sequestration of anthocyanins in the vegetative tissue of Arabidopsis has not been determined. This is critical since Arabidopsis can form a variety of modified cyanidin-type anthocyanins that are not found in seeds, but are found in other tissues and organs including stems and leaves^30^. In this study, using C3G as a model substrate, we demonstrate that anthocyanin uptake by vacuolar membrane-enriched vesicles isolated from Arabidopsis cell cultures occurs through both an ABC transporter and an H^+^-antiporter mechanism. Given the finding that VvABCC1 can serve as an anthocyanin/GSH co-transporter^26^ and that the ABC-transporters are generally better characterized in Arabidopsis than MATE transporters, we decided to examine which specific AtABCC transporter is involved in the vacuolar uptake of C3G. The two best characterized AtABCC transporters include AtABCC1 and AtABCC2 and both can be functionally expressed in yeast^31–33^. Both have been shown to transport a variety of substrates and are expressed in all Arabidopsis tissue examined^32^ including cell suspension cultures (Bio-Analytic Resource, ePlant Gene Expression Tool, www.bar.utoronto.ca). From *in vitro* C3G uptake assays with membrane vesicles isolated from yeast expressing AtABCC2, we demonstrated that, like VvABCC1^26^, AtABCC2 was a C3G/GSH co-transporter and transport did not depend on the formation of an anthocyanin-glutathione conjugate. We also showed that, in addition to anthocyanins, AtABCC2 could transport flavone and flavonol glucosides. Besides the biochemical studies, we also performed ligand docking to a homology model of AtABCC2 and investigated the putative binding sites of C3G and GSH through site-directed mutagenesis and functional studies. These studies revealed the close proximity of the C3G and GSH binding sites in AtABCC2 and identified residues important for ligand recognition and transport activity.

## Results

### C3G transport by vacuolar membrane-enriched vesicles from *Arabidopsis thaliana*

**Figure 2:**
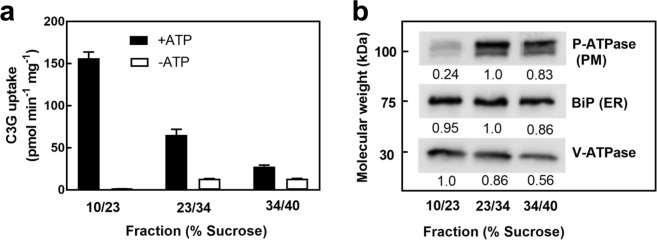
Uptake of C3G by Arabidopsis membrane vesicle fractions from discontinuous sucrose gradients. C3G uptake in the presence or absence of MgATP was determined in membrane vesicles collected from the interface between the indicated sucrose concentrations (**a**). Values shown are the means of three replicates ± SD. The enrichment of various membrane vesicles in each fraction was determined through western blots (**b**) using primary antibodies against the H^+^-ATPase (plasma membrane marker, PM), BiP luminal-binding protein (endoplasmic reticulum marker, ER), and the V-ATPase (vacuolar marker). Numbers under each blot represent relative pixel intensities of the bands in a row. The full-length blots are shown in Supplementary Fig. S3.

Previously it has been shown that transport competent vacuolar membrane-enriched vesicles can be isolated from Arabidopsis cell cultures and fractionated on discontinuous sucrose density gradients^14,34,35^. The vacuolar membrane enriched vesicles are collected from the interface between the 10% and 23% (10/23) sucrose gradients while the plasma membrane-enriched vesicles are collected at the interface between the 34% and the 40% (34/40) sucrose gradients^14,36^. A 23/34% fraction is typically added between the 10/23% and the 34/40% sucrose gradients^14,36^. Examination of C3G uptake activity in each of these fractions showed that the vesicles isolated from the 10/23% interfaced had the highest ATP-dependent uptake of C3G (Fig. 2a). This increase in uptake activity correlated with the vacuolar membrane enrichment as demonstrated by the increase in the immunodetection of the V-ATPase (vacuolar membrane marker; Fig. 2b). The immunodetection of H^+^-ATPase (plasma membrane marker) was low in the 10/23% fraction and high in the 23/34% and 34/40% fractions, while that of BiP (ER membrane marker) was unchanged across the three collected fractions (Fig. 2b). Therefore, MgATP-dependent C3G uptake activity was associated with the vacuolar membrane-enriched fraction from Arabidopsis cell cultures and this fraction was used in subsequent experiments.

There are generally two solvents that have been used to wash anthocyanins off of filters for HPLC analysis following transport assays. The first involves 50% methanol^6^ and the second involves 50% methanol/1% HCl^26^. To test the difference, we added a known amount of C3G in our assay media to the filters and washed the filters with either 50% methanol or 50% methanol/1% HCl. The samples were then analyzed by HPLC. Filters washed with 50% methanol as used in our study resulted in 83% recovery following HPLC analysis and filters washed with 50% methanol/1% HCl resulted in 82% recovery following HPLC analysis. These high recovery rates are virtually identical. In addition, we incubated C3G in either 50% methanol or 50% methanol/1% HCl at the concentrations used in our study and looked for degradation. However, the amounts recovered following HPLC were identical regardless of the incubation solution. In addition, the variation observed with our replicates was not correlated with the storage time (two days or less) of the filter extracts and we never observed any evidence of degradation during storage of the filter extracts.

**Figure 3:**
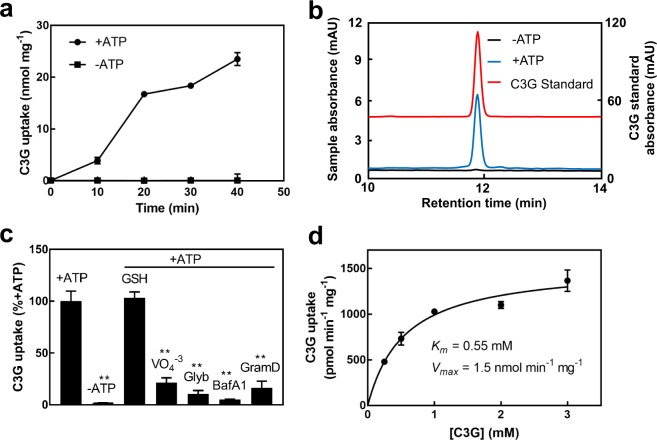
Uptake of C3G by Arabidopsis vacuolar membrane-enriched vesicles. (**a**) Time-dependent uptake of C3G by Arabidopsis vacuolar membrane-enriched vesicles in the presence or absence of MgATP. Values shown are the means of three replicates ± SD. Error bars that are not visible are located within the symbol marking the data point. (**b**) HPLC analysis of C3G uptake by Arabidopsis vacuolar membrane-enriched vesicles. Black line, −MgATP; blue line, +MgATP (left vertical axis); red line, C3G standard (right vertical axis). Though the scale is accurate, base lines have been shifted to allow for separation of HPLC traces. (**c**) Effect of GSH, vanadate, glybenclamide, bafilomycin A_1_, and gramicidin D on the uptake of C3G by Arabidopsis vacuolar membrane-enriched vesicles. GSH (5 mM) and vanadate (VO_4_^−3^; 1 mM) were dissolved in water. Bafilomycin A_1_ (BafA1; 0.4 μM), glybenclamide (Glyb; 150 µM), and gramicidin D (GramD; 5 µM) were dissolved in DMSO. All assay mixtures contained equivalent amounts of DMSO. The C3G uptake in the presence of 3 mM MgATP (173 pmol min^−1^ mg^−1^) was used as the control value and set at 100%. Other values were normalized to the control value and are the means of three replicates ± SD. Asterisks indicate a statistically significant decrease from the control value. **P < 0.01. (**d**) Effect of C3G concentration on uptake into Arabidopsis vacuolar membrane-enriched vesicles. Uptake rates in the presence of 3 mM MgATP at each C3G concentration were corrected for uptake rates during assays performed in the absence of MgATP. Values shown are the means of three replicates ± SD. Apparent *K*_*m*_ and *V*_*max*_ values were calculated as 0.55 mM and 1.5 nmol min^−1^ mg^−1^, respectively.

In the presence of MgATP, the uptake of C3G into Arabidopsis vacuolar membrane-enriched vesicles increased with time up to 40 min (Fig. 3a). In the absence of MgATP, the uptake of C3G was nearly undetectable and did not increase over time (Fig. 3a). The standard reaction time used for all subsequent C3G uptake assays with Arabidopsis vacuolar membrane-enriched vesicles was 15 min.

The HPLC retention time of C3G before addition to the uptake assay media (C3G standard) was 11.8 min (Fig. 3b). Following the uptake assays, the C3G that was washed off of the filters eluted as a single peak with the same HPLC retention time as the C3G standard (Fig. 3b). The retention time of C3G was the same regardless of whether or not the assay included MgATP (Fig. 3b). Therefore, C3G remained unaltered following uptake into the Arabidopsis vacuolar membrane-enriched vesicles.

Since the presence of MgATP resulted in an increase in the uptake of C3G by Arabidopsis vacuolar membrane-enriched vesicles, the next goal was to determine the mechanism of this uptake. Directly energized MgATP dependent uptake would likely involve an ABC transporter-type mechanism. Vanadate is a phosphoryl transition-state analog known to be a strong inhibitor of proteins that form a phosphoenzyme intermediate, including ABC transporters. Glybenclamide is a sulfonylurea derivative that effectively inhibits ABC transporters, especially subclass C (ABCC) transporters^37^. GSH is known to stimulate the ABCC transporter-mediated uptake of some organic compounds^27–29^. Indirectly energized MgATP dependent uptake would likely involve an antiporter that couples the movement of the substrate with that of a proton. A proton gradient would be formed in the presence of MgATP by the tonoplast localized V-type H^+^-ATPase. Bafilomycin A_1_ is a specific inhibitor of V-type H^+^-ATPases^38^ and gramicidin D is a cation-selective ionophore that would disrupt the pH gradient that would otherwise be formed in the presence of MgATP. The ABC transporter inhibitors vanadate (1 mM) and glybenclamide (150 µM) resulted in strong inhibition of C3G uptake (78.5% and 89% inhibition, respectively; Fig. 3c). Bafilomycin A_1_ (0.4 µM) and gramicidin D (5 µM) also resulted in strong inhibition of C3G uptake (95% and 83% inhibition, respectively; Fig. 3c). The presence of GSH (5 mM) had no effect on C3G uptake (Fig. 3c).

In Arabidopsis vacuolar membrane-enriched vesicles, the MgATP-dependent uptake of C3G exhibited Michaelis-Menten-type saturation kinetics with C3G concentration with apparent *K*_*m*_ and *V*_*max*_ values of 0.55 mM and 1.5 nmol min^−1^ mg^−1^, respectively (Fig. 3d).

### C3G transport by AtABCC2

**Figure 4:**
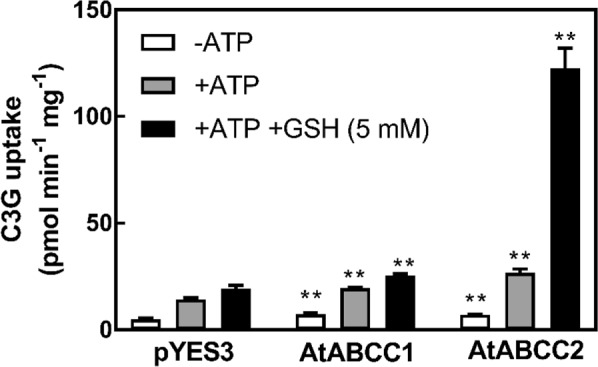
Uptake of C3G by membrane vesicles isolated from yeast strain DTY168 transformed with pYES3 (empty vector), pYES3-AtABCC1, or pYES3-AtABCC2. Gramicidin D (5 µM) was included in all assays to inhibit any endogenous H^+^ gradient-mediated uptake. When included, the concentrations of MgATP and GSH were 3 mM and 5 mM, respectively. The values shown are the means of three replicates ± SD. Asterisks indicate statistically significant differences when compared with the corresponding empty vector (pYES3) control. **P < 0.01.

In light of the recent finding that an ABCC transporter from grapevine (VvABCC1) is capable of C3G and GSH co-transport^26^, we examined the possibility that one of the known vacuolar membrane localized AtABCC transporters would also be capable of C3G and GSH co-transport. The two best characterized AtABCC transporters in Arabidopsis are AtABCC1 and AtABCC2^31–33^. In the absence of MgATP, yeast transformed with pYES3, pYES-AtABCC1, or pYES3-AtABCC2 had minimal C3G uptake activity, which increased slightly in the presence of MgATP (Fig. 4). The most dramatic increase in C3G uptake was observed in the presence of both MgATP and GSH with vesicles isolated from the pYES3-AtABCC2 transformed yeast. This uptake activity was 4.6-fold greater than that in the presence of MgATP alone and 6.3-fold higher than the C3G uptake activity in the presence of MgATP and GSH observed with membrane vesicles isolated from pYES3 transformed yeast. Comparable increases in C3G uptake activity were not observed for membrane vesicles isolated from yeast transformed with pYES3-AtABCC1. In addition, the uptake of cyanidin (aglycone) in the presence or absence of MgATP or GSH by membrane vesicles isolated from yeast expressing AtABCC2 was undetectable.

**Figure 5:**
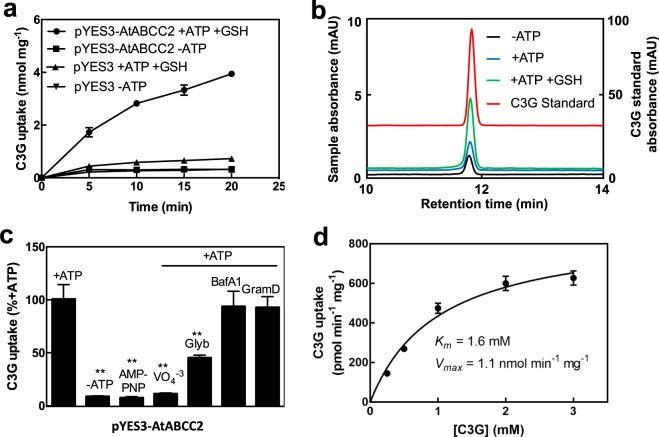
Uptake of C3G by AtABCC2. (**a**) Time-dependent uptake of C3G by membrane vesicles isolated from yeast strain DTY168 transformed with either pYES3 (empty vector) or pYES3-AtABCC2. Gramicidin D (5 µM) was included in all assays to inhibit any endogenous H^+^ gradient-mediated uptake. When included, the concentrations of MgATP and GSH were 3 mM and 5 mM, respectively. The values shown are the means of three replicates ± SD. Error bars that are not visible are located within the symbol marking the data point. (**b**) HPLC analysis of C3G uptake by membrane vesicles isolated from yeast strain DTY168 transformed with pYES3-AtABCC2. Black line, −MgATP; blue line, +MgATP; green line, +MgATP +GSH (left vertical axis); red line, C3G standard (right vertical axis). Though the scale is accurate, base lines have been shifted to allow for separation of HPLC traces. (**c**) Effect of AMP-PNP, vanadate, glybenclamide, bafilomycin A_1_, and gramicidin D on the uptake of C3G by membrane vesicles isolated from yeast strain DTY168 transformed with pYES3-AtABCC2. Assays mixtures contained GSH (5 mM) and equivalent amounts of DMSO. AMP-PNP (3 mM) and vanadate (VO_4_^−3^; 1 mM) were dissolved in water. Glybenclamide (Glyb; 150 µM), bafilomycin A_1_ (BafA1; 0.4 μM), and gramicidin D (GramD; 5 µM) were dissolved in DMSO. The C3G uptake in the presence of 3 mM MgATP (100.5 pmol min^−1^ mg^-1^) was used as the control value and set at 100%. Other values were normalized to the control value and are the means of three replicates ± SD. Asterisks indicate a statistically significant decrease from the control value. **P < 0.01. (**d**) Effect of C3G concentration on uptake by membrane vesicles isolated from yeast strain DTY168 transformed with pYES3-AtABCC2. Gramicidin D (5 µM) and GSH (5 mM) were included in all assays. Uptake rates in the presence of 3 mM MgATP at each C3G concentration were corrected for uptake rates during assays performed in the absence of MgATP. Values shown are the means of three replicates ± SD. Apparent *K*_*m*_ and *V*_*max*_ values were calculated as 1.6 mM and 1.1 nmol min^−1^ mg^−1^, respectively.

In the presence of MgATP and GSH, the uptake of C3G into yeast vesicles expressing AtABCC2 increased with time up to 20 min (Fig. 5a). Unless otherwise indicated, all other assays using yeast vesicles containing heterologously expressed AtABCC2 were conducted for 15 min in the presence of both MgATP and GSH.

Following the uptake assays with membrane vesicles isolated from yeast expressing AtABCC2, the C3G that was washed off of the filters eluted as a single peak with the same HPLC retention time as the C3G standard (Fig. 5b). The retention time of C3G was the same regardless of whether or not the assay included MgATP or GSH (Fig. 5b). Therefore, as with the assays conducted with Arabidopsis vacuolar membrane-enriched vesicles, the C3G remained unaltered following transport and there was no evidence of the formation of a C3G-glutathione conjugate even in uptake assays that included GSH.

In order to determine the mechanism of C3G uptake into the yeast vesicles expressing AtABCC2, we examined the uptake in the presence of various transport inhibitors. C3G uptake was completely abolished in the presence of vanadate (1 mM) and strongly inhibited (55% inhibition) by glybenclamide (150 µM), two known inhibitors of ABCC transporters (Fig. 5c). The nonhydrolyzable analog of ATP, adenosine 5′-β,γ-imino)triphosphate (AMP-PNP), could not substitute for ATP in the transport assays indicating that ATP hydrolysis is required for transport (Fig. 5c). The V-type H^+^-ATPase inhibitor, bafilomycin A_1_ (0.4 µM) or the cation-selective ionophore, gramicidin D (5 µM) had no effect on C3G uptake (Fig. 5c). Therefore, transport was not dependent on the formation of an H^+^ gradient. The pattern of inhibition observed is consistent with C3G transport by AtABCC2 occurring through an ABC-transporter-mediated mechanism.

The MgATP-dependent uptake of C3G into yeast vesicles expressing AtABCC2 exhibited Michaelis-Menten-type saturation kinetics with C3G concentration with apparent *K*_*m*_ and *V*_*max*_ values of 1.6 mM and 1.1 nmol min^−1^ mg^−1^, respectively (Fig. 5d).

### Substrate specificity of AtABCC2

**Figure 6:**
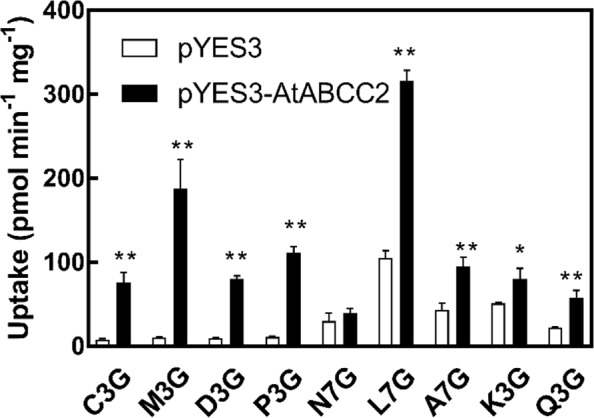
Uptake of C3G and other flavonoid-glycosides by membrane vesicles isolated from yeast strain DTY168 transformed with either pYES3 (empty vector) or pYES3-AtABCC2. The flavonoids were dissolved in DMSO and added to a final concentration of 100 µM. Gramicidin D (5 µM) and GSH (5 mM) were included in all assays. The data shown represent the uptake rates in the presence of MgATP (3 mM) minus the uptake rates in the absence of MgATP. The values shown are the means of three replicates ± SD. Asterisks indicate statistically significant differences when compared with the corresponding empty vector (pYES3) control. **P < 0.01; *P < 0.05. C3G, Cyanidin 3-*O*-glucoside; M3G, Malvidin 3-*O*-glucoside; D3G, Delphinidin 3-*O*-glucoside; P3G, Pelargonidin 3-*O*-glucoside; N7G, Naringenin 7-*O*-glucoside; L7G, Luteolin 7-*O*-glucoside; A7G, Apigenin 7-*O*-glucoside; K3G, Kaempferol 3-*O*-glucoside; Q3G, Quercetin 3-*O*-glucoside.

To determine the substrate specificity of AtABCC2, we isolated membrane vesicles from yeast expressing AtABCC2 and conducted uptake assays in the presence of four anthocyanins (C3G, M3G, D3G, and P3G), a flavanone glucoside (N7G), two flavone glucosides (L7G and A7G), and two flavonol glucosides (K3G and Q3G; Fig. 1). A significant increase in the uptake activity above that observed for control assays (vesicles from yeast transformed with empty vector) was observed for all substrates tested except N7G (Fig. 6). The greatest relative increase above the control values was observed for all four anthocyanins; with M3G exhibiting the greatest fold increase. However, the greatest overall uptake activity was observed for L7G. Therefore, it appears that AtABCC2 can transport all of the anthocyanins tested and a variety of other flavonoid glucosides.

### L7G transport by AtABCC2

**Figure 7:**
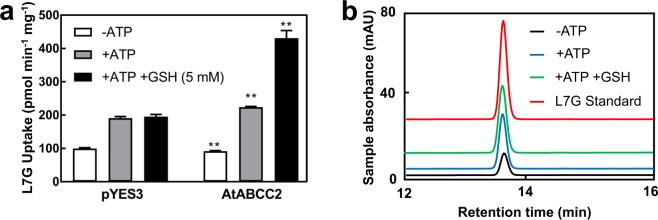
Uptake of L7G by AtABCC2. (**a**) Uptake of L7G by membrane vesicles isolated from yeast strain DTY168 transformed with either pYES3 (empty vector) or pYES3-AtABCC2. Gramicidin D (5 µM) was included in all assays in order to inhibit any endogenous H^+^ gradient-mediated uptake. When included, the concentrations of MgATP and GSH were 3 mM and 5 mM, respectively. The values shown are the means of three replicates ± SD. Asterisks indicate statistically significant differences when compared with the corresponding empty vector (pYES3) control. **P < 0.01. (**b**) HPLC analysis of L7G uptake by membrane vesicles isolated from yeast strain DTY168 transformed with pYES3-AtABCC2. Black line, −MgATP; blue line, +MgATP; green line, +MgATP + GSH; red line, L7G standard. Though the scale is accurate, base lines have been shifted to allow for separation of HPLC traces.

Since a high AtABCC2-mediated uptake activity in the presence of MgATP and GSH was observed with L7G (Fig. 6), we investigated the effects of MgATP and GSH on L7G transport by vesicles isolated from yeast expressing AtABCC2. Compared to the control, the addition of MgATP significantly increased L7G uptake (Fig. 7a). The addition of both MgATP and GSH further increased the L7G uptake of vesicles isolated from yeast expressing AtABCC2, but no further increase was seen for membrane vesicles isolated from the control. The addition of MgATP and GSH increased the uptake activity of vesicles from yeast expressing AtABCC2 about 2-fold compared to the addition of MgATP alone and 4.7-fold compared to the uptake observed in the absence of MgATP (Fig. 7a). Following the uptake assays with membrane vesicles isolated from yeast expressing AtABCC2, the L7G that was washed off of the filters eluted as a single peak with the same HPLC retention time as the L7G standard (Fig. 7b). The retention time of L7G was the same regardless of whether or not the assay included MgATP or GSH (Fig. 7b). Therefore, L7G, like C3G, remained unaltered following transport.

### C3G promoted transport of [^3^H]GSH by AtABCC2

In the absence of C3G or L7G, we were able to detect the MgATP-dependent uptake of [^3^H]GSH by yeast vesicles expressing AtABCC2 (Table 1). Though the MgATP-dependent uptake of [^3^H]GSH was not increased in the presence of L7G, the inclusion of C3G resulted in a statistically significant 3.6-fold increase in [^3^H]GSH uptake. Neither C3G nor L7G had any effect on the MgATP-dependent uptake of [^3^H]GSH by membrane vesicles isolated from yeast transformed with the empty vector (pYES3; Table 1). Therefore, AtABCC2 functions as an anthocyanin/GSH co-transporter, but not as a flavone/GSH co-transporter. It should also be noted that C3G did not promote the uptake of [^3^H]GSH by vacuolar membrane-enriched vesicles isolated from Arabidopsis cell cultures.

**Table 1:** Effects of C3G and L7G on the uptake of [^3^H]GSH by AtABCC2 heterologously expressed in yeast (DTY168).

Vesicles	Assay Conditions			
	+MgATP, −C3G	+MgATP, +C3G	+MgATP, −L7G	+MgATP, +L7G
	(pmol min^−1^ mg^−1^)			
pYES3/DTY168	86.3 ± 55.4	83.6 ± 15.9	59.4 ± 31.5	35.9 ± 30.3
pYES3-AtABCC2/DTY168	210.6 ± 5.22	753.0 ± 141.1**	230.3 ± 61.6	205.1 ± 37.2

The final concentration of [^3^H]GSH in the assay medium was 5 mM (0.25 µCi/µmole; 0.25 µCi per assay), and when included, the final concentrations of C3G and L7G in the assay medium were 0.5 mM. All values were corrected for uptake in the absence of MgATP. The values shown represent the means of three independent uptake experiments ± SD. Asterisk indicates statistically significant difference between the +MgATP, −C3G and +MgATP, +C3G uptake values (**P < 0.01).

### Putative binding sites of C3G and GSH in AtABCC2

To determine the putative binding sites of C3G and GSH, we combined molecular modeling, ligand docking, site-directed mutagenesis and *in vitro* assays of mutants. Through a Basic Local Alignment Search Tool (BLAST; https://blast.ncbi.nlm.nih.gov/) search of AtABCC2 against the Protein Data Bank (PDB) database, we found four crystal structures of AtABCC2 homologues: PDB ID 5UAK, 5W81, 5WUA and 6BHU, with sequence identity to AtABCC2 of 28, 31, 32 and 38%, respectively. As the crystal structures represent different conformational states of the ABC transporters, ligand docking depended on the substrate cavity space associated with the transporter state. Thus, ligand docking in AtABCC2 homology models based on PDB ID 5W81 and 6BHU failed to provide reasonable solutions.

**Figure 8:**
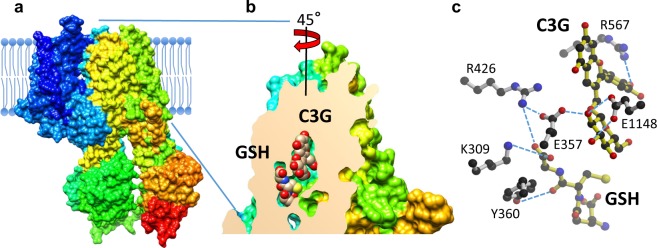
C3G and GSH docking to the homology model of AtABCC2 based on PDB ID 5WUA. (**a**) Overall 3D structure in rainbow color scheme, starting at the N-terminus in blue and ending at the C-terminus in red. (**b**) C3G and GSH binding sites in the transmembrane domain, shown in a view rotated by 45 degrees compared with (**a**). (**c**) Hydrogen bond interactions between AtABCC2 residues and ligands (C3G and GSH).

**Figure 9:**
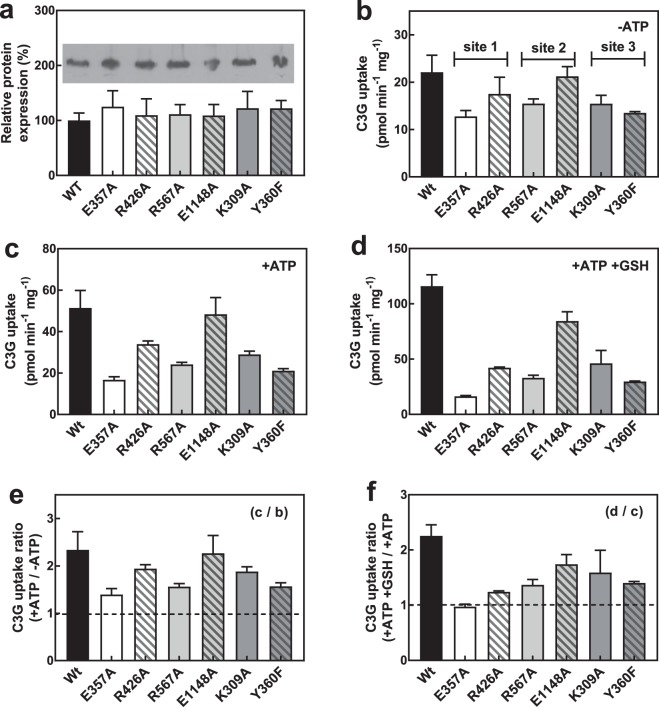
AtABCC2 mutants in C3G and GSH binding sites. (**a**) Protein expression of mutants relative to wild-type. The western blot (inset) is one of three individual replicates. The full-length blot is shown in Supplementary Fig. S4. (**b**) C3G uptake in the absence of MgATP. Sites 1, 2 and 3 refer to residues making hydrogen bonds with both C3G and GSH, only C3G, and only GSH, respectively. (**c**) C3G uptake in the presence of 3 mM MgATP. (**d**) C3G uptake in the presence of 3 mM MgATP and 5 mM GSH. (**e**) C3G uptake ratio with/without 3 mM MgATP. (**f**) C3G uptake ratio with/without 5 mM GSH, in the presence of 3 mM MgATP (transport activation by GSH). Values shown are the means of three replicates ± SD.

On the other hand, the AtABCC2 homology model based on PDB ID 5WUA showed two distinct binding sites for C3G and GSH with adequate physicochemical properties (Fig. 8). The binding site for C3G was found in the same location in the AtABCC2 homology model based on PDB ID 5UAK, whereas the GSH binding site overlapped with the C3G site in the latter homology model. Overlapped binding sites for C3G and GSH would suggest competition between these ligands and are incompatible with the functional data that shows that GSH activates C3G uptake. Therefore, for the structural analysis we used the AtABCC2 homology model based on PDB ID 5WUA. To validate the identified C3G and GSH binding sites, we mutated residues that putatively interacted with these ligands and examined the activity of the mutants *in vitro* (Fig. 9). We distinguish three sites composed of residues that interact with: both C3G and GSH (site 1), only C3G (site 2), and only GSH (site 3). E357 is within hydrogen bond distance from both C3G and GSH and can also interact with R426, the other residue close to both C3G and GSH (Fig. 8c). K309 and Y360 make hydrogen bond interactions with C3G, while E1148 and R567 interact with the glucosyl and cyanidin moieties of C3G. The mutants expressed well (Fig. 9a) and the measured transport activity was normalized based on the level of protein expression as indicated by western analysis (Fig. 9b–d). All six mutants had impaired transport activity and GSH activation, compared to the wild-type protein (Fig. 9d). E357A (site 1) mutation abolished transport activity irrespective of MgATP or GSH addition. The next group of mutations with the most impact on transport activity (ranging between 17% and 26% of wild-type activity) were R426A (site 1), R567A (site 2), and Y360F (site 3; Fig. 9d). The mutation with the lowest effect on transport activity was E1148A (retained 33% wild-type activity) despite two possible hydrogen bond interactions with the glucosyl group of C3G. Irrespective of whether the mutants were in the C3G or GSH binding site, they all showed significant reduction in GSH transport activation, at a level of 0–60% of wild-type (Fig. 9f). On the other hand, MgATP transport activation of the mutants (i.e. transport activity in the presence versus absence of MgATP) was not significantly affected (level of 30–95% of the wild-type, Fig. 9e).

### Putative binding sites of L7G in AtABCC2

The Ligand docking of L7G to an AtABCC2 model based on PDB ID 5WUA showed that the L7G binding site spans both the C3G and GSH binding sites identified above (See Supplementary Fig. S1). Thus, L7G and GSH cannot both bind to the active site simultaneously, consistent with the finding that AtABCC2 is not a flavone/GSH co-transporter.

## Discussion

We demonstrate that the vacuolar membrane-enriched fraction from Arabidopsis cell cultures transports C3G in an MgATP-dependent manner. This transport was strongly inhibited by known inhibitors of ABC transporters and H^+^-antiporters, suggesting that both an ABC transporter and an H^+^-antiporter are involved in C3G transport. Surprisingly, in the presence of the ABC transporter inhibitors, the H^+^ antiporter-mediated uptake did not compensate for the inhibition of the ABC transporter and vice versa. Both an ABC transporter and H^+^-antiporter were also thought to be involved in the uptake of abscisic acid glucosyl ester (ABA-GE) by Arabidopsis vacuoles^39^ and salicylic acid 2-O-β-D-glucose by Arabidopsis vacuolar membrane-enriched vesicles^14^. The pattern of inhibition with C3G may suggest that not only are two transport mechanisms involved, but that there might be some interaction where the inhibition of one affects the activity of the other. There are numerous examples of ABC proteins interacting with and regulating other transporters^40–47^. However, further research will be necessary to determine if there is an interaction between tonoplast localized ABCC transporters and H^+^-antiporters during the vacuolar sequestration of anthocyanins.

Given that an ABC transporter appeared to be involved in the uptake of C3G by Arabidopsis vacuolar membrane-enriched vesicles, it was of interest to determine which specific ABC protein might be involved. The vacuolar membrane of Arabidopsis contains 10 ABC subclass C members (AtABCC1-8, 10 and 14)^48^ and the AtABCC proteins are the predominate ABC transporters localized to the vacuolar membrane^49^. Within this group, AtABCC1 and 2 were the first plant ABCC transporters identified and are the best characterized in terms of their *in vitro* transport activity^31–33,39,50,51^. Interestingly, both were also thought to be capable of transporting the glutathione conjugate of C3G (C3G-GS)^31,32^. However, these results were questioned since other investigators^10^ were unable to replicate the chemical formation of the [^3^H]C3G-GS substrate. It was suggested that the severe chemical conditions (e.g. high pH, heat, long incubation time) used for the synthesis of [^3^H]C3G-GS from C3G and [^3^H]GSH resulted in the formation of oxidized [^3^H]GSH ([^3^H]GSSG) rather than [^3^H]C3G-GS^10^. In any case, C3G-GS was tested as a possible substrate for AtABCC1 and AtABCC2 following the discovery of the involvement of both GST enzymes (BZ2) and ABCC transporters (ZmABCC3 and 4) in the vacuolar sequestration of anthocyanins in corn^9,25^. The closest ZmABCC3 homolog in Arabidopsis is AtABCC10^25,26^. However, to the best of our knowledge, AtABCC10 has not been linked to anthocyanin transport in Arabidopsis and it has not been shown to transport anthocyanins in heterologous expression systems. More recently, it has been demonstrated that VvABCC1 is involved in the vacuolar accumulation of anthocyanins in grape berry^26^. The *in vitro* transport of anthocyanins was strictly dependent on GSH though no anthocyanin-GS conjugate was formed; rather, VvABCC1 was a co-transporter for both anthocyanins and GSH. The obvious involvement of ABCC transporters in the vacuolar sequestration of C3G and the finding that VvABCC1 was capable of anthocyanin and GSH co-transport, prompted us to reexamine the ability of AtABCC1 and 2 to transport C3G. As with VvABCC1, we were able to demonstrate that AtABCC2, but not AtABCC1, could transport C3G in a GSH dependent manner. During our *in vitro* assays, there was no chemical change in C3G following transport ruling out the formation of a C3G-GS conjugate. Rather, AtABCC2, like VvABCC1, was an anthocyanin/GSH co-transporter since C3G would promote the uptake of GSH and vice versa. This is the first demonstration that an ABCC transporter from the model organism Arabidopsis is capable of C3G/GSH co-transport.

The MgATP-dependent transport of C3G by Arabidopsis vacuolar membrane-enriched vesicles exhibited Michaelis-Menton-type saturation kinetics (K_m_ for C3G of 0.55 mM) as did the MgATP-dependent uptake of C3G in the presence of GSH by AtABCC2 expressed in yeast (K_m_ for C3G of 1.6 mM). Our calculations (see Supplementary Information) indicate that the cyanidin-type anthocyanin concentration can reach 34 µM in cells of Arabidopsis leaves under typical soil-grown conditions and 0.38 mM in the cytosol of leaf cells experiencing stress conditions. This cytosolic concentration under stress conditions begins to approach the K_m_ values for both AtABCC2 expressed in yeast and the vacuolar membrane-enriched vesicles. It should also be noted, that the cyanidin-type anthocyanins in Arabidopsis leaves are decorated with xylosyl, malonyl, coumaryl, and sinapoyl moieties^52^. These naturally occurring cyanidin-type anthocyanins are the more likely natural substrates of AtABCC2 and the other possible transporters in the vacuolar membrane and may be transported at a higher affinity than observed for the model substrate C3G. This may be similar to what was observed for MtMATE2, which had a kinetic preference for malonylated anthocyanins over the non-malonylated glycosides^18^.

In addition to anthocyanins, MtMATE2 has also been shown to transport a variety of other flavonoids^18^. Therefore, we were interested in determining if this was also the case for AtABCC2. We demonstrated that AtABCC2 could transport all anthocyanins examined in the presence of MgATP and GSH. We did not observe any significant uptake of the aglycones. Therefore, at a minimum, glucosylation is a requirement for transport. In addition to anthocyanins, AtABCC2 had significant uptake capacity for flavones (L7G and A7G) and flavonols (K3G and Q3G), but not the flavonone N7G. When MtMATE2 was expressed in yeast it was capable of significant anthocyanin (C3G) transport as well as transport of flavone glucosides (A7G and L7G) and a flavanone glucoside (N7G). However, *in vitro* MtMATE2 did not transport flavonol glucosides. It is significant that AtABCC2 can transport flavonol glucosides, since in addition to cyanidin-type anthocyanins, quercetin and kaempferol-type flavonols are also prevalent in the vegetative tissue of Arabidopsis^30,52–54^. The presence of two transporter types (ABCC and MATE transporters) in the vacuolar membrane of Arabidopsis may extend the range of the types of flavonoids that are transported into the vacuole. However, it remains to be determined if the H^+^-antiporter-mediated uptake of C3G by Arabidopsis vacuolar membrane-enriched vesicles involves a MATE transporter.

Given the relatively high uptake rate for L7G by AtABCC2, we decided to further explore the transport mechanism of this substrate. Even though L7G is not found in Arabidopsis^52^, it does have roles in other plants including alfalfa where it may be a *nod*-gene inducing compound in *Rhizobia meliloti*^55,56^. Dietary Luteolin from plants has also been shown to function in animals as an anti-oxidant, anti-inflammatory, and anti-cancer agent^57^. In species that produce luteolin glucosides, ABCC transporters may be involved in the vacuolar sequestration and thus the regulation of the activity of this flavonoid. Interestingly, though ABCC2 appeared to be a cotransporter of C3G and GSH, this effect was not observed with L7G; while GSH enhanced L7G transport, L7G did not affect GSH transport. Ligand docking studies showed that the L7G binding site overlaps with the GSH and C3G binding sites, supporting the finding that AtABCC2 is not a L7G/GSH co-transporter. Regardless of the assay conditions, L7G was not altered after transport indicating that a GSH conjugate was not formed. A multisite model for AtABCC2 has been proposed that includes semi-autonomous transport pathways and distinct binding sites^33^. GSH has been shown to stimulate the uptake of β-estradiol 17-(β-D-glucuronide) (E_2_17βG), but E_2_17βG did not promote GSH transport. Given the similarities, it is possible that the transport of L7G may occur through the same site on AtABCC2 as E_2_17βG.

While we could demonstrate that ABCC2 expressed in yeast was capable of C3G and GSH cotransport, we did not see a difference in transport rates or inhibitor sensitivities between vacuolar membrane-enriched vesicles isolated from wild-type or *atabcc2* mutants (see Supplementary Fig. S2). In addition, knock-out mutants of AtABCC2 did not show any flavonoid-related phenotypes^58^ and AtABCC2 expression is not influenced by regulators of anthocyanin formation in Arabidopsis^52^. Therefore, it is possible that in addition to AtABCC2, other transporters (e.g. ABCC and/or MATE transporters) are also involved in the vacuolar uptake of flavonoids including anthocyanins.

GSH stimulation of C3G transport by yeast vesicles expressing AtABCC2 was not replicated in Arabidopsis vacuolar membrane-enriched vesicles. It is possible that endogenous factors modulating the activity of C3G uptake by ABCC transporters co-purify with the isolated vacuolar membrane-enriched vesicles. These factors would be absent in the yeast used as a heterologous expression system for AtABCC2. The addition of GSH might then substitute for the missing factors in yeast. One possible factor might involve the membrane bound immunophilin-like protein TWISTED DWARF1 (TWD1). TWD1 has been shown to interact with both AtABCC1 and 2 and modulate the activity of organic anion transport by isolated vacuoles in a manner similar to that observed for both glutathione conjugates and GSH^59^.

Though there is now evidence that the uptake of anthocyanins by at least two plant ABCC transporters (i.e. AtABCC2 and VvABCC1; 27.2% sequence identity at the amino acid level) can be enhanced by the presence of GSH, the transport mechanism at the protein level has not been explored until now. As indicated by the molecular modeling analysis of AtABCC2 and supported by mutagenesis studies, the central cavity in the membrane part of the transporter contains the C3G and GSH binding sites in close proximity to each other. Mutants of residues from these ligand binding sites show variable degrees of impairment in transport activity and GSH activation; coupling between ATP hydrolysis and transport activity in all the mutants is not significantly affected, though slightly lower. Mutations of R426 and E357 residues at the binding sites boundary reduce transport activity significantly (R426, 19% of the wild-type activity) or completely (E357). They are probably residues important for coordinating and organizing the ligand binding as they can interact with either ligand as well as with each other, with E357 playing a more critical role. Single-site mutations in residues that interact only with one of the ligands (R567 and E1148 for C3G or Y360 and K309 for GSH), decrease transport activity by 30 to 60% of the wild-type, indicating that there is no particular residue essential for ligand binding and that ligand recognition is distributed among the residues forming the binding site. Nevertheless, the larger side-chain residues do seem more important probably because they impose steric constraints besides providing hydrogen-bond interactions to ligand binding. E1148, which comes from the C-terminus half of the transporter, is predicted to make hydrogen bonds with two hydroxyl groups in the glucosyl moiety of C3G, however its mutation still retains 33% of wild-type C3G uptake activity. Most residues in the C3G and GSH binding sites belong to the N-terminus half domain; C-domain residues may move in and out of the ligand binding site in response to ATP hydrolysis. Thus, E1148 may not be involved in the initial binding of C3G to AtABCC2, explaining the modest effect its mutation has on transport activity. In AtABCC2, K309 and Y360 in the GSH binding site are more than 8 Å away from where C3G putatively binds, however their mutations affect C3G uptake even when GSH is absent. This coupled with the proximity of the C3G and GSH binding sites, and with the finding that mutants in the C3G binding site (R567A and E1148A) impair GSH transport activation, suggest that C3G and GSH are not independently recognized, rather C3G and GSH influence each other’s binding. Whether this is the case for other GSH-dependent ABC transporters remains to be established. In the crystal structure of a GSH-binding ABC transporter, Atm1^60^, the GSH binding site is located similarly as in the AtABCC2 model, close to the inner membrane surface in the cavity, but the amino acids involved in GSH binding (R280, R284, S394, and D398) are not conserved in AtABCC2. This suggests that while the recognition of GSH may vary, the mechanism by which GSH enhances transport of substrates in ABC transporters may be more general.

In conclusion, we suggest that AtABCC2 along with possibly other ABCC proteins and MATE transporters is involved in the vacuolar transport of anthocyanins and other flavonoids in the vegetative tissue of Arabidopsis. The identification of an ABCC transporter in Arabidopsis that is capable of the *in vitro* anthocyanin transport and the wealth of genetic resources available for this model organism should allow for a more complete investigation of the role of ABCC transporters, GSH, GST (TT19), TWD1, and possible ABCC/MATE protein-protein interactions in the vacuolar sequestration of anthocyanins. In addition, through molecular modeling analysis, site-directed mutagenesis, and functional transport studies of AtABCC2, we suggest that the C3G and GSH binding sites are in the central cavity of the membrane localized portion of the transporter and are in close proximity to one another such that ligand binding is coordinated. These studies also allowed us to identify amino acid residues important for ligand recognition and transport activity. Understanding the mechanistic details of GSH transport activation and substrate recognition in AtABCC2 will provide essential insights into the functional versatility of ABC transporters.

## Materials and Methods

### Chemicals

Flavonoids were either purchased from Alkemist labs (Costa Mesa, CA, USA) or MilliporeSigma (St. Louis, MO, USA). [*glycine*-2-^3^H]GSH was purchased from American Radiolabeled Chemicals (St. Louis, MO, USA). Zymolyase 20 T was purchased from AMS Biotechnology (Abingdon, UK). All other chemicals were purchased from MilliporeSigma or Fisher Scientific (Pittsburgh, PA, USA).

### Arabidopsis Cell Cultures and Preparation of Vacuolar Membrane-enriched Vesicles

Arabidopsis cell cultures were initiated and maintained as described by Vaca *et al*.^14^. Vacuolar membrane-enriched vesicles from Arabidopsis cell suspension cultures were prepared through differential and density gradient centrifugation as described by Rea *et al*.^61^ using the step gradients described by Rea and Turner^36^ and modified for use with Arabidopsis cell cultures as described by Vaca *et al*.^14^. The protein concentration was estimated by the method of Bradford^62^ using BSA as a protein standard.

### Heterologous expression of AtABCC1 and AtABCC2 in *Saccharomyces cerevisiae*

The *S. cerevisiae ycf1*Δ strain DTY168 (*MATα his6 leu2-3,-112, ura 3-52 ycf1::hisG*), the yeast-*Escherichia coli* shuttle vector designated pYES3, and the constructs pYES3-AtABCC1 (formally pYES3-AtMRP1) and pYES3-AtABCC2 (formally pYES3-AtMRP2) were obtained from Dr. Philip A. Rea (University of Pennsylvania)^31,32^. For the studies involving site-directed mutagenesis, pYES3-AtABCC2 was further modified to have an octa-histidine tag at the N terminus: MGSSHHHHHHHHSSGLVPRGSH. For this ATGGGCAGCAGCCATCACCATCACCATCACCATCATCACCATAGCAGCGGTCTGGTTCCGCGTGGTAGCCAT along with 693 bp of the 5′ end of AtABCC2 DNA^32^ was commercially synthesized (GenScript, Piscataway, NJ). Both the synthesized DNA and the plasmid were cut and ligated with the NcoI and SphI restriction endonucleases. To generate mutant proteins, site-directed mutagenesis was performed on the plasmid constructs of the wild-type protein^63^. All DNA constructs were verified by sequencing. The yeast strain DTY168 was transformed with vectors by the LiOAc/polyethylene glycol method^64^, and selected for uracil prototrophy on AHC media^65^ supplemented with tryptophan (32 µM).

### Preparation of yeast membrane vesicles

The preparation of yeast membrane vesicles for *in vitro* uptake experiments was performed essentially as describe previously^65–68^ with some modifications. AHC media with tryptophan (200 mL) was inoculated with a single yeast colony and incubated for 24 h at 30 °C with shaking (150 rpm). The 200 mL culture was then diluted to 1 liter with fresh AHC plus tryptophan medium and incubated 24 h with shaking at 30 °C. The cells were collected by centrifugation (1200 *g* for 10 min), washed twice with water, and suspended in 50 mL of 1.1 M sorbitol, 20 mM Tris-HCl (pH 7.6), 1 mM DTT containing 37 mg zymolyase 20 T. The suspension was incubated for 90 min at 30 °C with gentle shaking (75 rpm). The resulting spheroplasts were pelleted by centrifugation (1200 *g* for 10 min) and resuspended into 25 mL of ice cold homogenization buffer (1.1 M glycerol, 5 mM Tris-EGTA, 1% [w/v] sodium ascorbate, 50 mM Tris-MES [pH 7.4], 1 mM PMSF, 1 µg/mL leupeptin). After disruption in a 50-mL glass dounce homogenizer, the cell debris was removed by centrifugation at 4000 *g* for 10 min. The resulting pellet was resuspended in 10 mL of homogenization buffer, disrupted in a dounce homogenizer and centrifuged as described above. The two supernatants were pooled and centrifuged at 100,000 g for 45 min. The pellet was resuspended in 1.3 mL buffer (1.1 M glycerol, 1 mM Tris-EGTA, 2 mM DTT, 50 mM Tris-MES [pH 7.4], 1 µg/mL leupeptin) to an A_600_ of approximately 4.0 and stored in liquid nitrogen for no more than 24 h before they were used for uptake assays. The protein concentration was estimated by the method of Bradford^62^ using BSA as a protein standard.

### Transport assays

Measurement of flavonoid uptake into Arabidopsis vacuolar membrane-enriched vesicles and yeast membrane vesicles was performed essentially as described by Li *et al*.^34^ and Bartholomew *et al*.^69^ in 200 µL reaction volumes. The standard uptake assay contained 25 mM Tris-MES (pH 8.0), 0.4 M sorbitol, 50 mM KCl, 3 mM MgSO_4_, 0.1% (w/v) bovine serum albumin, 10 mM creatine phosphate, 3 mM ATP, 16 units mL^−1^ creatine kinase and various concentrations of substrates (100 μM for the standard assay). Gramicidin D (5 µM) was included when the assays were conducted with yeast membrane vesicles in order to inhibit any endogenous H^+^ gradient-mediated uptake. The reaction was initiated by the addition of either 24 μL (ca. 5.5 mg protein/mL) of vacuolar membrane-enriched vesicles or 24 µL yeast membrane vesicles (ca. 10 mg/mL) and was allowed to proceed at 25 °C for the indicated times (15 min for the standard assay). Reactions were terminated by the addition of 1 mL of ice cold wash buffer (25 mM Tris-MES [pH 8.0], 0.4 M Sorbitol) followed by vacuum filtration of the suspension through prewetted Millipore HA 0.45 µm cellulose nitrate filters. The filters were washed twice with 1 mL of ice cold wash buffer and air-dried. Filters were then transferred to 20 mL screw cap glass vials containing 1 mL 50% (v/v) methanol. The filters were extracted for 1 h at room temperature with shaking (140 rpm). The extracts (50 µL) were loaded onto a 150 × 4.6 mm Alltima 5 µ C18 HPLC column (Grace Discovery Sciences, Deerfield, IL, USA) connected to a Waters (Milford, MA, USA) 1525 binary HPLC pump and a Waters 2489 UV/Vis detector. The column was eluted at a flow rate of 1 ml min^−1^ with a linear gradient from 95% perchloric acid (0.6% v/v) and 5% methanol to 20% perchloric acid (0.6% v/v) and 80% methanol in 20 min. Data were collected at the following wavelengths; cyanidin, C3G, D3G, M3G and P3G, 535 nm; A7G, 334 nm; K3G, 350 nm; luteolin, 348 nm; L7G, 353 nm; N7G, 283 nm; and Q3G, 358 nm. When included, the assays contained the following final concentrations of inhibitors/activators: 0.4 µM Bafilomycin A_1_, 1 mM Vanadate, 5 µM gramicidin D, 150 µM glybenclamide, 3 mM AMP-PNP, and 5 mM GSH. All inhibitors/activators were dissolved in DMSO (which was less than 1% [v/v] of the final assay volume) except for GSH, vanadate, and AMP-PNP which were dissolved in water. The assays used to determine the effects of flavonoids on the uptake of [^3^H]GSH were conducted as described above except that all assays included 5 mM NH_4_Cl and the radioactivity on the filters was determined through liquid scintillation counting rather than HPLC. The final concentration of [^3^H]GSH in the assay medium was 5 mM (specific activity of 0.25 µCi/µmol; 0.25 µCi per 200 µL assay) and the final concentration of flavonoids was 0.5 mM.

### Western blot analysis

Membrane vesicles from Arabidopsis cell cultures were collected from the interface of each of the three step gradients described by Rea and Turner^36^. Primary antibodies against the V-ATPase (vacuolar marker), H^+^-ATPase (plasma membrane marker), and BiP luminal-binding protein (endoplasmic reticulum marker) were obtained from Agrisera (Vännäs, Sweden) and western blot analysis was conducted as described by Vaca *et al*.^14^. Relative pixel intensities were determined with the Image Studio operating software of the Li-Cor Odyssey Fc imager (Lincoln, NE). Western blot analysis for His-tagged wild-type and mutant AtABCC2 proteins expressed in yeast was performed with RGS-His HRP Conjugate according to the manufacturer’s instructions (Qiagen, Germantown, MD). As expression level of wild-type and mutant AtABCC2 proteins varied, the transport activity was normalized. To determine the relative expression of AtABCC2 proteins, yeast cells were cultured as described in the “Preparation of yeast membrane vesicles” section, and cell pellets were processed as described by Zhang *et al*.^70^. In short, for each AtABCC2 protein the cell pellet corresponding to 10 O.D. units (one O.D. unit defined as 1 ml of cell culture at OD_600nm_ 1.0) was resuspended in 1 ml of 2 M Lithium Acetate, incubated on ice for 5 minutes, recollected by centrifugation, resupended in 0.5 ml water, combined with 0.5 ml 0.8 M NaOH, incubated on ice for 5 minutes, and recollected by centrifugation. This final pellet was resuspended in 30 µl water and combined with 100 µl 2X SDS-PAGE sample buffer. The SDS-PAGE gel (10% polyacrylamide) was loaded with 15 µl sample/lane and used for Western blot analysis. Quantification of the bands in the Western blot was performed with ImageJ^71^.

### Computational modeling of the C3G, L7G and GSH binding sites in an AtABCC2 homology model

The AtABCC2 3D structure is unknown, so AtABCC2 homologues with available 3D structures were identified with a BLAST search against the PDB database. PDB ID 5UAK, 5W81, 5WUA and 6BHU were used to generate homology models for AtABCC2 with Molecular Operating Environment (MOE) software (https://www.chemcomp.com). The C3G, L7G and GSH binding sites in the AtABCC2 models were determined with MOE SiteFinder and Dock functions, using a set of possible C3G, L7G and GSH conformations generated with MOE Conformation Search function. The virtual docking of C3G, L7G and GSH in the AtABCC2 homology models was carried out with Triangle Matcher parameters retaining 100 poses and London dG rescoring. Identified binding sites for CG3, L7G and GSH were evaluated based on physicochemical properties of residues interacting with CG3, L7G and GSH.

## Supplementary information

Supplementary information

## Data Availability

All data generated or analyzed during this study are included in this published article and its Supplementary information files.
