# Supplementary material for: Transport of Anthocyanins and other Flavonoids by the Arabidopsis ATP-Binding Cassette Transporter AtABCC2
Source: Sci Rep. 2019 Jan 24;9:437. doi: 10.1038/s41598-018-37504-8 (PMC6345954; doi:10.1038/s41598-018-37504-8)

Supplementary information

Transport of Anthocyanins and other Flavonoids by the Arabidopsis ATP-Binding Cassette Transporter AtABCC2

Claire E. Behrens[1], Kaila E. Smith[1], Cristina V. Iancu[2], Jun-yong Choe[2]*, and John V. Dean[1]*

Authors' Affiliations:

[1]Department of Biological Sciences, DePaul University, Chicago, 60614, USA

[2]Department of Biochemistry and Molecular Biology, Rosalind Franklin University of Medicine and Science, North Chicago, 60064, USA

***Corresponding Authors**

J. V. Dean, jdean@depaul.edu

J. Choe, junyong.choe@rosalindfranklin.edu

Calculations used to determine cytosolic and vacuolar concentrations of cyanidin-type anthocyanins in Arabidopsis leaf cells.

Using values reported previously[1], the cumulative totals for cyanidin-type anthocyanins in soil-grown wild-type Arabidopsis leaves and a *pap1-D* mutant (overexpressor of the *PAP1* gene which encodes a MYB transcription factor) can reach levels of 11.5 and 129.2 nmol g^{-1} FW, respectively. It is possible to speculate that 90% of the anthocyanins are localized in the vacuole and 10% in the cytosol based on the distribution of other glucose conjugated plant compounds such as ABA-GE[2]. If we also assume that the volume of the cytosol is ~34 µL g^{-1} FW[3], then we can calculate a vacuolar volume (~136 µL g^{-1} FW) and protoplast volume (~170 µL g^{-1} FW) based on the assumption that the vacuole represents 80% of the protoplast volume. Given these assumptions, we can calculate a cytosolic and vacuolar cyanidin-type anthocyanin concentration of 34 µM and 76 µM, respectively, in Arabidopsis leaves under typical soil-grown conditions. In leaves experiencing stress conditions (*pap1-D* mutant), the cytosolic and vacuolar concentrations could reach 0.38 mM and 0.86 mM, respectively.

1. Tohge, T. *et al.* Functional genomics by integrated analysis of metabolome and transcriptome of Arabidopsis plants over-expressing an MYB transcription factor. *Plant J.* **42**, 218-235 (2005).

2. Bray, E.A. & Zeevaart, J.A.D. The compartmentation of abscisic acid and β-D-glucopyranosyl abscisate in mesophyll cells. *Plant Physiol.* **79**, 719-722 (1985).

3. Raichaudhuri, A. *et al.* Plant vacuolar ATP-binding cassette transporters that translocate folates and antifolates *in vitro* and contribute to the antifolate tolerance *in vivo*. *J. Biol. Chem.* **284**, 8449-8460 (2009).

Supplementary Fig. S1. C3G, L7G and GSH docking to the homology model of AtABCC2 based on PDB ID 5WUA. C3G (magenta), L7G (cyan) and GSH (yellow) binding sites are shown in the surface capping model. L7G binding site spans that of GSH.

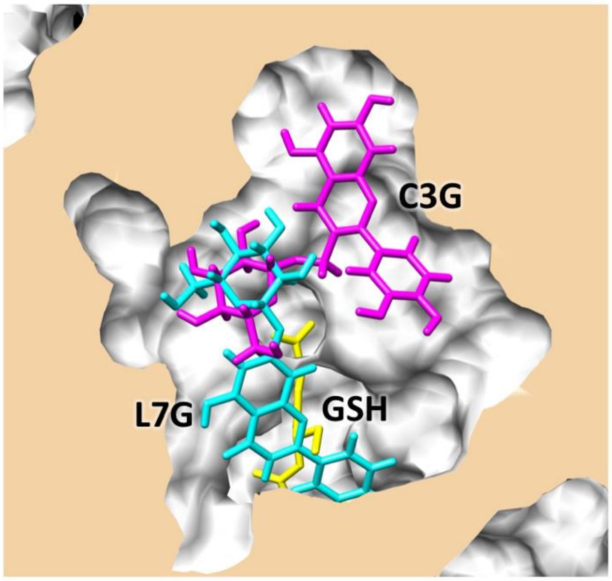

Supplementary Fig. S2. Effects of MgATP, vanadate and gramicidin D on the uptake of C3G by Arabidopsis tonoplast-enriched vesicles isolated from wild-type (Col-0) or *atabcc2* cell cultures. Vanadate (VO_4^{-3}; 1 mM) was dissolved in water. Gramicidin D (5 µM) was dissolved in ethanol. The uptake assays were conducted as described in the Materials and Methods in the presence or absence of 3 mM MgATP and all assays mixtures contained equivalent amounts of ethanol. Values shown are the means of three replicates ± SD. Asterisks indicate a statistically significant difference. *, $P < 0.05$.

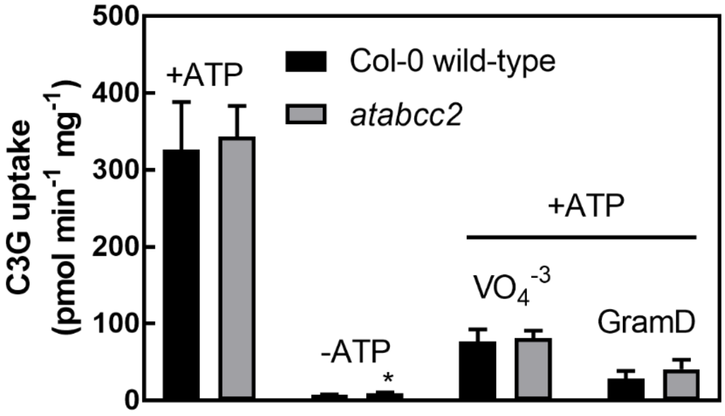

Supplementary Fig. S3. Full-length western blotting gels of membrane vesicle fractions from sucrose gradients (Manuscript Figure 2b). The enrichment of various membrane vesicles in each fraction was determined through western blots using primary antibodies against the (a) H^+-ATPase (plasma membrane marker, PM), (b) BiP luminal-binding protein (endoplasmic reticulum marker, ER), and the (c) V-ATPase (vacuolar marker).

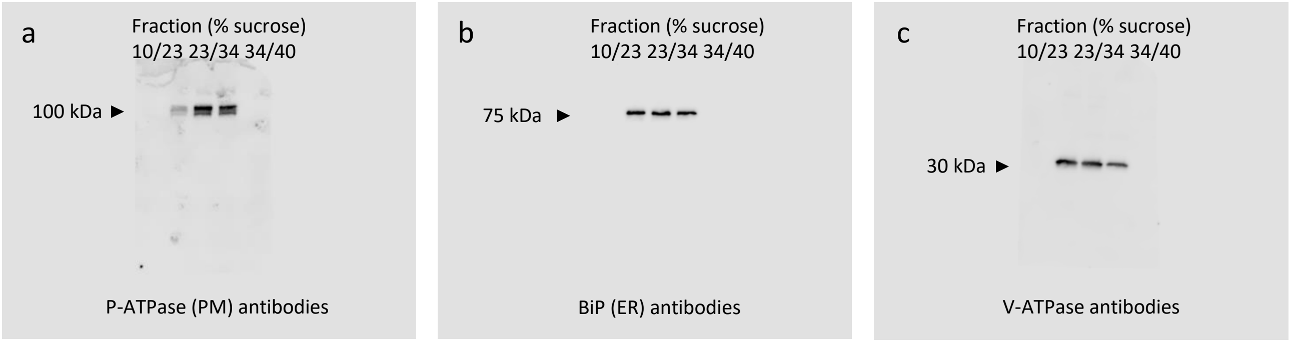

Supplementary Fig. S4. Full-length western blotting gel of AtABCC2 expression (Manuscript Figure 9a). Theoretical molecular weight of AtABCC2 (1623 amino acid residues) is 182 KDa. AtABCC2 migrates 30-40% further than a typical soluble protein of the same molecular weight.

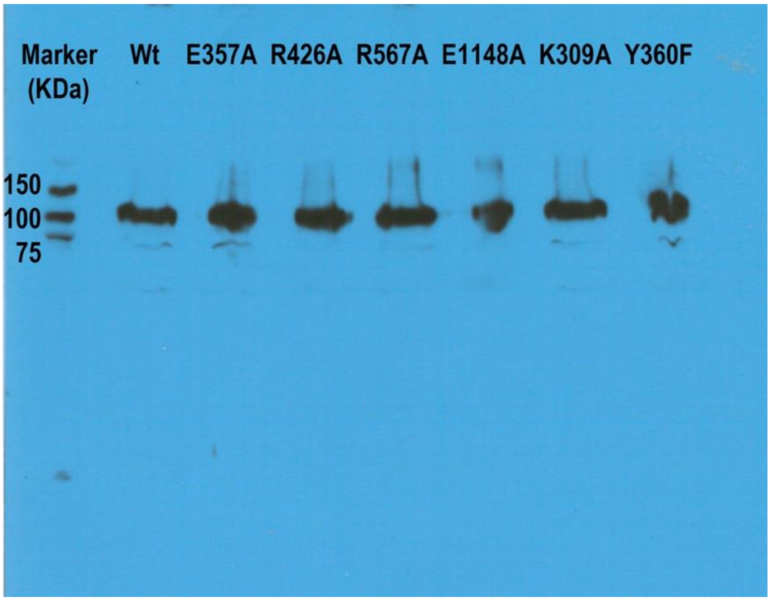

Supplement: Supplementary file 1 — Supplementary information [file 41598_2018_37504_MOESM1_ESM.pdf]
